# Observations on the Angustura Bark

**Published:** 1790

**Authors:** G. Wilkinson

**Affiliations:** Surgeon at Sunderland, Member of the Royal College of Surgeons, and honorary Member of the Chirurgo-physical Society of Edinburgh


II. *Obſervations on the Anguſtura Bark. Communicated in a Letter to Dr.* Simmons, *F. R. S. by Mr.* G. Wilkinſon, *Surgeon at Sunderland, Member of the Royal College of Surgeons, and honorary Member of the Chirurgo-phyſical Society of Edinburgh.*

AFTER having peruſed the very favourable accounts given of this bark, in the London Medical Journal *, by Drs. Ewer and Williams, I was induced to ſend for ſome to Meſſrs. Taylor and Davy; ſince

* Vol. X. p. 154, 158.

which time I have had frequent opportunities of trying its effects in various cafes, but more particularly in fome of thofe wherein it is fuppofed the good effects of the Peruvian bark chiefly prevail, and where it would feem that the cortex Anguſturæ has greatly the advantage.

In the accounts alluded to*, which feem indeed only general with refpect to its effects, no mention is made of the quantity of bark ufed in the decoction as a fomentation; neither are we told the proportion internally exhibited in the Madeira wine.

At firft I was fomewhat at a lofs in what manner to prefcribe this bark, not only with refpect to its dofe, but likewife the method in which it would be moft proper to adminifter it in different cafes.

On a little reflection it occurred to me that a fmaller quantity of the Anguftura bark would be more likely to produce effects on the conftitution than the Peruvian, more particularly as I found its tafte remarkably bitter, with a confiderable degree of pungency or warmth, of a refinous quality, though fomewhat difagreeable

* Vol. X. p. 156, 161.

in in its flavour: its bitternefs feemed to me to refemble that of the colombo root, and of this opinion were fome medical gentlemen to whom I have fhewn it.

With thefe fentiments I fet about confidering the various modes in which it might be adminiftered, viz. thofe of infufion, decoction, tincture, electuary, and powder *.

What

* The firft of thefe I prepare by infufing half an ounce of grofs powder of the Anguftura bark, and one drachm and a half of caffia lignea in powder, in a pint of boiling water, for about two hours. The dofe is from two to four fpoonfuls as occafion may require.

The fame quantity of the bark boiled with about twenty ounces of water, for fifteen minutes, makes the decoction; to eight ounces of which I fometimes add one fcruple of powder of nutmeg, as I find it improves the medicine greatly.

One ounce of the bark digefted in a pint of proof fpirit gives out nearly the whole of its virtues. This I fometimes add occafionally to the infufion or decoction, in the proportion of one ounce to eight ounces, or half an ounce to four or fix ounces, with a few drops of the compound tincture of lavender.

In the form of an electuary it is very grateful, and fits eafy on the ftomach, prepared thus: — Anguftura bark, in fine powder, half an ounce; powder of cinnamon, one drachm and a half, or two drachms; conferve of Seville orange peel, one ounce; fyrup of ginger, or fimple fyrup, a fufficient quantity to form an electuary.

The

What has been obferved by Dr. Williams * and Mr. Brande †, of this bark's being lefs apt, than the Peruvian bark, to difagree with the ftomach, is perfectly juft, and the following cafe in fome degree evinces the truth of the obfervation.

This was the firft cafe that came under my obfervation: it was an irregular intermittent. The patient had taken the Peruvian bark in various forms before I was confulted, and I myfelf gave it to her in decoction, with the addition of a ftrong infufion of caffia buds and aromatic tincture; neverthelefs it difagreed with her ftomach, and went off by ftool. I then had recourfe to the Anguftura bark, which agreed well with her ftomach; but we were foon obliged to difcontinue it, her complaint proving to be a pulmonary affection, which afterwards terminated fatally.

The powder I have generally exhibited, without any addition to it, in water, or any other fuitable vehicle. In fome inftances I have added to it powder of cinnamon in the proportion of one drachm to three drachms of the powder of the bark. None of thefe additions, I am perfuaded, tend in any degree to influence the effects of the medicine.

* Vol. X. page 159.

† See page 40.

A cafe

A cafe of periodical head-ach occurred, which attacked the patient with great violence, and for which various remedies were prefcribed, as tincture of opium and antimonial wine, during the fit, and the Peruvian bark in confiderable quantities in the intervals of the paroxyfms, but without effect. The complaint, however, was removed by adminiftering fix or eight dofes, of ten grains each, of the Anguftura bark in powder, every three or four hours, in a glafs of white wine and water.

In two or three cafes of violent tooth-ach, attended with excruciating pain, a general affection of the fyftem, and evening paroxyfms refembling the fit of an ague, this bark has proved of fingular fervice. One of thefe, which had continued a long time, and for which the Peruvian bark had been given without effect, was cured by fix dofes of the Anguftura bark, each of which confifted only of fix grains of the powder, without any other addition.

Nothing, however, has furprifed me fo much as the effects of this bark as a febrifuge. In fome cafes of low, or what is termed nervous, fever, and in that fort of irregular intermittent, in children, ufually called worm fever, I have experienced wonderful effects from it. In thefe I have have given the infufion with the tincture, and that without any regard to intermiffions.

Two cafes of intermittents have occurred to me wherein the powers of this medicine feemed greatly fuperior to thofe of the Peruvian bark. One of thefe was a tertian, in a patient thirty-four years of age, a feafaring man, who had contracted it at Middleburgh in Zealand. The fits, which returned with great regularity, were very long and violent. He had likewife a fymptomatic jaundice, attended with coftivenefs and frequent ficknefs. After premifing an emetic, exhibiting a faline purge or two, and a bolus of calomel and rhubarb, he took fix dofes of the pure powder of Anguftura bark, each dofe containing ten grains. Thefe put a ftop to the diforder, and, by way of preventing a relapfe, fix powders more were given, which completed his cure.

The other cafe was a quotidian. The patient was a young man, nineteen years of age, who had taken feveral remedies prior to the ufe of the Anguftura bark, which was adminiftered to him in the form of an infufion, to fix ounces of which was added half an ounce of the tincture. Three fpoonfuls of this mixture were directed to be taken every three hours, when the fit was abſent. This quantity was repeated three times, and he was cured.

No opportunity has yet occurred for me to give it a trial in ſcrophula, mortification, or ſcorbutic caſes; yet I ſhould not be ſurpriſed at its producing very ſalutary effects in ſuch complaints.

In a caſe where an elderly man wounded the capſular ligament of the knee joint, and where the ſynovia was diſcharged from the wound, (and the patient having imprudently attempted to walk a day or two after the accident, the moſt violent ſymptoms took place, ſuch as a troubleſome hiccough, &c.) the Peruvian bark was given with opium, but the patient's ſtomach could by no means bear it. I then ſubſtituted in its ſtead the Anguſtura bark, in powder, in doſes of fifteen grains. In this form it agreed with his ſtomach, though the diſcharge from the wound and great irritation afterwards occaſioned his death.

Twice I have tried the infuſion in diarrhœas. In one of theſe caſes the patient voided a ſmall quantity of blood; but the good effect of the medicine was ſo ſudden that I was the more ſurpriſed at it, as, Mr. Brande juſtly ob-

ſerves*, this bark betrays no aſtringency to the taſte.

With reſpect to this bark as a tonic or ſtomachic, I can truly ſay it ſeems to me greatly preferable to the Peruvian. The frequent opportunities I have had of trying its effects in thoſe diſorders termed nervous, or, in other words, what is uſually called *dyſpepſia*, in moſt of which caſes there is conſtantly more or leſs of a morbid irritability of the ſtomach and inteſtines, induces me to recommend its uſe, more particularly as I have experienced its uſefulneſs in ſeveral inſtances of this ſort.

Neverthelefs I muſt remark, that in thoſe caſes where a quantity of bile or acidity prevails in the firſt paſſages, and the patient is ſubject to coſtiveneſs, I have generally preceded the exhibition of this medicine by an emetic, or by gentle laxatives, as magneſia, or rhubarb, and have accompanied its uſe with ſoluble tartar.

In ſhort, when it is conſidered that this bark produces ſuch deciſive effects, and that even in much ſmaller doſes than any other medicine of the ſame ſort, I flatter myſelf future experience

* See page 45.

will will be truly fenfible of its being a great acquifition to the materia medica.

Happy in having it in my power to corroborate Mr. Brande's obfervations in moft of thefe remarks, I muft beg leave moft heartily to recommend this bark to the farther notice of medical practitioners.

*Sunderland,*
Auguft 5, 1790.

